# Urease inhibitors: current advances, therapeutic potentials, and clinical challenges

**DOI:** 10.1080/17568919.2026.2684497

**Published:** 2026-06-15

**Authors:** Omer Habis Alzoubi, Almu’atasim Khamees, Mohammad Basil Alzu’bi, Sarah Feras Freihat, Aya Attar, Hanan Abo Sameed, Rawan Alzubi, Amen Ahed Alsamarah, Raghad Yousef Yassin, Khayry Al-Shami, Bahaa Al-Trad, Raed M. Al-Zoubi, Mazhar Salim Al Zoubi

**Affiliations:** a Department of Basic Medical Sciences, Faculty of Medicine, Yarmouk University, Irbid, Jordan; b Princess Basma Teaching Hospital, Ministry of Health, Irbid, Jordan; c Faculty of Pharmacy, Yarmouk University, Irbid, Jordan; d Department of Biomedical Sciences, College of Veterinary Medicine, King Faisal University, Al-Ahsa, Saudi Arabia; e Surgical Research Section, Department of Surgery, Hamad Medical Corporation, Doha, Qatar; f Department of Chemistry, Jordan University of Science and Technology, Irbid, Jordan; g Department of Biomedical Sciences, QU-Health, College of Health Sciences, Qatar University, Doha, Qatar

**Keywords:** Urease inhibitors, *Helicobacter pylori*, struvite kidney stones, hepatic encephalopathy, acetohydroxamic acid

## Abstract

Urease is a nickel-dependent metalloenzyme that catalyzes urea hydrolysis, generating ammonia and elevating local pH, a process implicated in the pathogenesis of *Helicobacter pylori*–associated peptic ulcer disease (PUD). To identify effective urease inhibitors, acetohydroxamic acid (AHA) remains the only FDA-approved agent, with clinical use constrained by toxicity. This review synthesizes current evidence on urease inhibition strategies, encompassing metal-based complexes, organic derivatives, natural products, peptide inhibitors, nanotechnology-enabled systems, and emerging gene-based approaches. Several candidates, including copper-based compounds and the natural alkaloid palmatine (PAL), demonstrate potent urease inhibition and favorable effects in preclinical models. However, translation to clinical practice is limited by safety concerns, instability, and a paucity of human trials. Advancing hybrid molecules, optimized delivery platforms, and combination therapies may enhance therapeutic efficacy. Rigorous clinical evaluation remains essential to establish urease inhibition as a viable strategy in urease-mediated diseases.

## GRAPHICAL ABSTRACT

**Figure:**
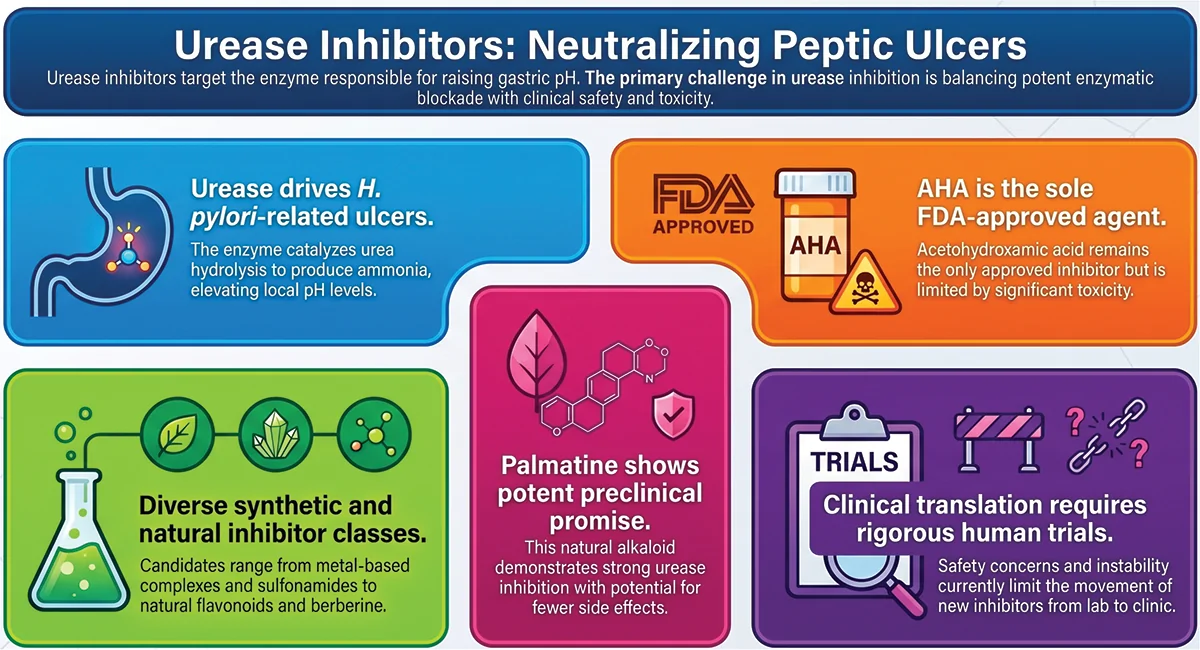

## 1. Introduction

**Figure 1.**
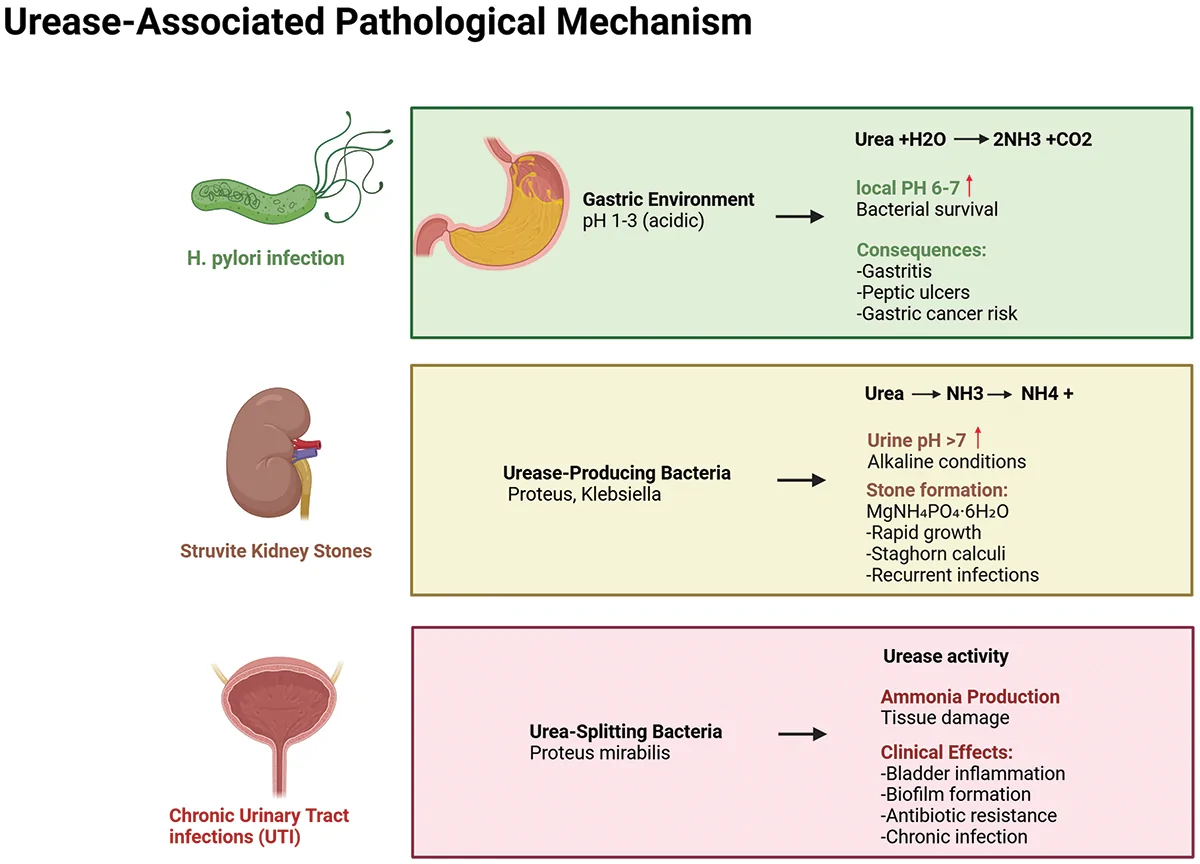
Illustration of the three major pathological mechanisms mediated by bacterial urease activity in distinct anatomical sites: *H. pylori* infection in the gastric environment. Urease-producing bacteria (*Proteus*, *Klebsiella*) in struvite kidney stones. Urea-splitting bacteria (*Proteus mirabilis*) in chronic urinary tract infections (UTIs). Created in Biorender. Al-Zoubi, RM. (2025) http://Biorender.com?zwkojeh.

Ureases are high-molecular-weight, nickel-containing metalloenzymes (biocatalysts containing one or more transition-metal ions; in this case, nickel) that belong to the amidohydrolase and phosphotriesterase families [1]. Urease is a heteromeric molecule with three subunits: α, β, and γ. The active site of urease is located in the α subunit, which contains two Ni_2_+ centers (Ni1 and Ni2) that are crucial to the catalyst’s mechanism. One nickel ion activates urea, while the other activates nucleophilic water molecules [2]. Urea is synthesized primarily in the liver via the urea cycle enzymes and is then eliminated through fluids (especially urine) [1,3]. Elevation of the PH of the surrounding environment due to the basic qualities of the ammonia produced, making the urease a major contributor in the pathogenesis of some diseases, such as hepatic encephalopathy (HE), peptic ulcer caused by *Helicobacter Pylori* (*H. pylori*) and *Klebsiella*, *Proteus mirabilis*-mediated urolithiasis (Figure 1) [4,5].

**Figure 2.**
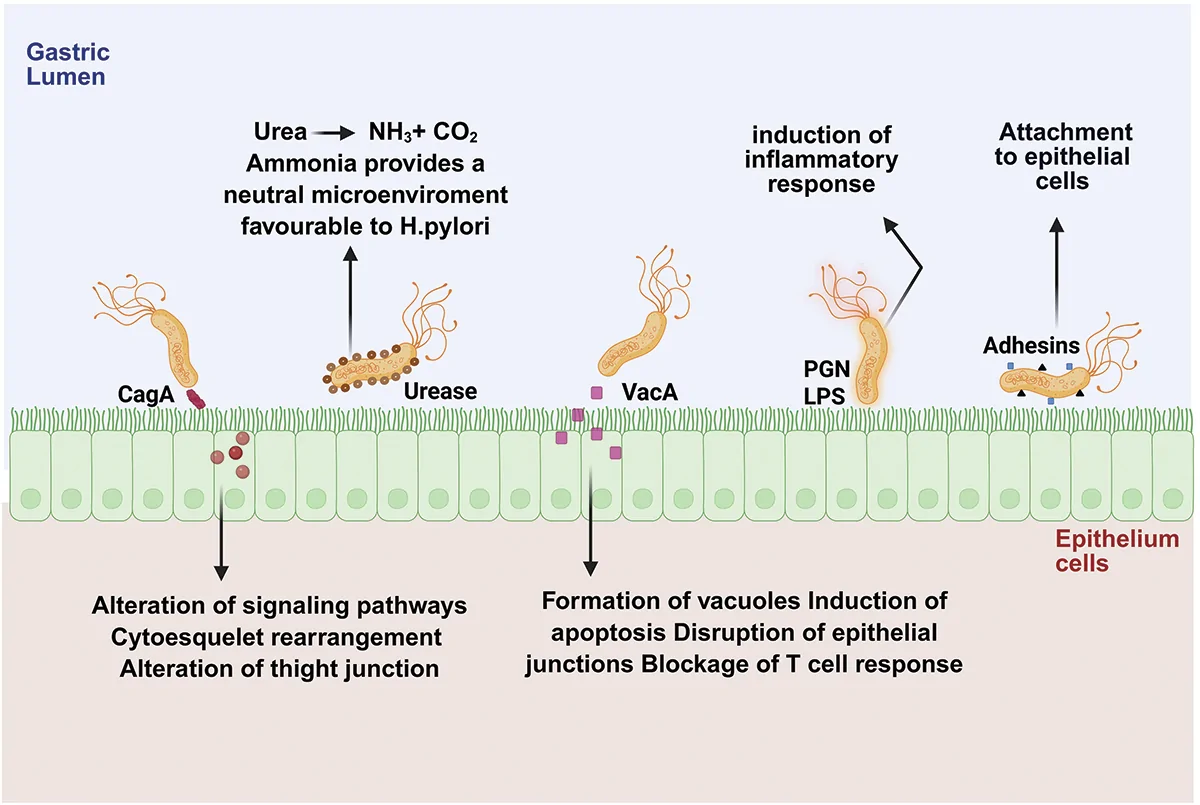
Representation of the *H.pylori* virulence factors and gastric epithelial interactions. Urease enzyme hydrolyzes urea into ammonia (NH_3_) and carbon dioxide (CO_2_), creating a neutral microenvironment that protects the bacterium from gastric acid. CagA protein is injected into epithelial cells via a type IV secretion system, triggering cytoskeletal rearrangement, altered signaling pathways, and disruption of tight junctions, while VacA toxin induces vacuole formation, apoptosis, epithelial junction disruption, and impaired T cell responses. Bacterial surface components, including peptidoglycan (PGN), lipopolysaccharide (LPS), and adhesins mediate attachment to epithelial cells and trigger inflammatory responses that contribute to chronic gastritis and disease progression. Created in Biorender. Al-Zoubi, RM. (2025) http://Biorender.com?zwkojeh.

Naturally, urease is not found in the human body; rather, it is found in numerous bacteria, such as *Proteus mirabilis, Staphylococcus saprophyticus, Helicobacter pylori*, and other microorganisms, as well as in some fungi, soil enzymes, algae, and certain invertebrates. Therefore, its enzymatic activity has a toxic effect on the human body [6]. It is also useful for taxonomic identification, diagnosis, and tracking the effectiveness of treatment (Figure 2) [7].

The ammonia produced by *H. pylori* urease neutralizes stomach acid and damages the gastric mucosa. Leading to inflammation, disruption of the epithelial barrier, and increasing the risk of developing gastric ulcers and gastric cancer [8,9]. In addition, relying on urease activity, *Proteus mirabilis* is a common cause of catheter-associated urinary tract infections (CAUTIs). The alkaline conditions facilitate the colonization and rapid spread of *Proteus mirabilis* within the urinary tract [10]. Patients with cirrhosis may have elevated blood ammonia levels due to *H. pylori* and other microbial infections. In these patients, ammonia, a neurotoxin that penetrates the blood–brain barrier, has a role in neurological and cognitive impairment (Figure 1). Antibiotics such as rifaximin may reduce the number of bacteria that produce urease, thereby lowering ammonia production and improving HE outcomes. It has been demonstrated that treating *H. pylori* in certain situations reduces ammonia levels and improves clinical outcomes [11,12].

The current review aims to evaluate the efficacy of urease inhibitors in the treatment of urease-mediated diseases. Also, address the most up-to-date available urea blockers and the clinical trials that have assessed their safety, reliability, and efficacy. In addition to discovering possible synergies with other treatments for urease-mediated pathologies, such as triple and quadruple treatment regimens in *H. pylori* treatment. Moreover, we review trials to assess the efficacy and reliability of these agents, not only *in vitro* but also *in vivo*.

## 2. Urease pathophysiology

### 2.1. H. pylori in peptic ulcer disease

**Figure 3.**
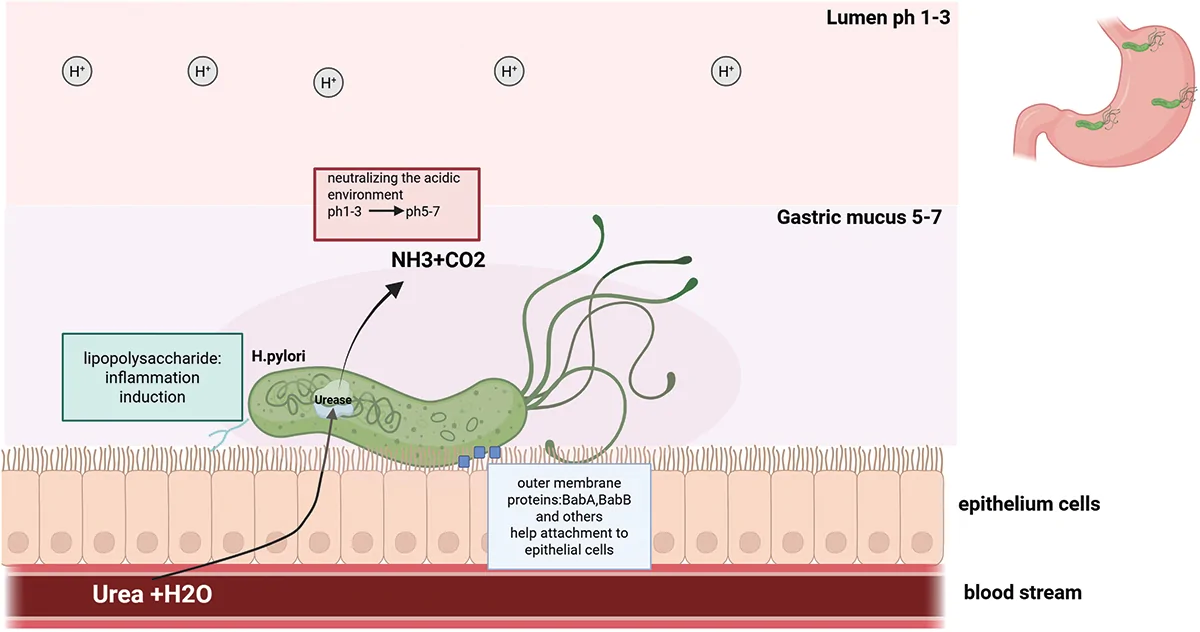
This figure illustrates the mechanism by which *H. pylori* survives and colonizes the acidic gastric environment (lumen pH 1–3). The bacterium produces urease enzyme, which catalyzes the hydrolysis of urea (diffusing from the bloodstream through epithelial cells) into ammonia (NH_3_) and carbon dioxide (CO_2_), thereby neutralizing the surrounding acidic environment and elevating local pH from 1–3 to 6–7. This urease-mediated acid neutralization creates a protective microenvironment that enables bacterial survival, while bacterial surface components, including lipopolysaccharide, induce inflammation, and outer membrane proteins (BabA/BabB) facilitate attachment to epithelial cells, ultimately establishing persistent gastric colonization within the protective mucus layer (pH 5–7). Created in Biorender. Al-Zoubi, RM. (2025) http://Biorender.com?zwkojeh.

PUD is defined as a discontinuity of the GI tract lining layer due to gastric acid secretion and pepsin, which extends to the muscularis propria layer of the gastric epithelium [13]. Most cases of PUD are associated with *H. pylori* infection. The bacteria and their accompanying inflammation are believed to contribute to the pathophysiology of PUD [14], and gastric cancer [15]. *H. pylori* produces a 550 kDa, multimeric, nickel-containing urease that catalyzes the hydrolysis of urea to yield ammonia and carbonic acid (Figure 3). Urease is crucial for the survival and virulence of *H. pylori* and is important for colonization in the gastric mucosa by neutralizing gastric acid and providing ammonia for bacterial protein synthesis [7,16]. Moreover, urease plays a role in bacterial colonization, stimulation of bacterial nutrients, generation of the proton motive force, modulation of the host immune response, and activation of platelets and angiogenesis [17]. Ammonia, a byproduct of urea breakdown, is cytotoxic to the gastric epithelium; it also triggers a strong immune response during acute infection [18]. *H. pylori* detection tests in the gastric mucosa include rapid urease test on gastric biopsy or mucus and the urea breath test, which measures the change in isotope enrichment of ^13^C- or ^14^CO_2_ in breath following oral administration of labeled urea [19].

### 2.2. Hepatic encephalopathy (HE)

HE is a neuropsychiatric manifestation that can vary in severity from mild alterations in mental status to coma [20]. Ammonia, primarily produced in the colon by intestinal bacteria, is considered to be the primary toxin involved in the pathogenesis of HE [21]. Evstafeva et al. examined a hydroxamate-based urease inhibitor, 2-octynohydroxamic acid, for inhibition of bacterial urease, and showed low cytotoxicity and mutagenic potential and reduced ammonia in rodent model of liver disease; therefore, the researchers recommended the process of colon delivery of urease inhibitors as an admirable approach for treatment of HE [22].

### 2.3. Struvite stones of the kidney

Kidney stones (nephrolithiasis) are mineral deposits in the renal calyces and pelvis. These stones form when the urine becomes supersaturated with certain substances, most commonly calcium oxalate (60%), while other substances, such as magnesium and citrate, have an inhibitory effect on stone formation. Clinical presentation varies, as only 60% of patients are symptomatic. Kidney stones are highly prevalent, affecting approximately 14.8% of the general population, with a recurrence rate of 50% within the first 5 years after the initial stone formation [23,24]. Struvite stones are a less common type of stone that is believed to be caused by urease-producing bacteria associated with many complications, such as sepsis, and carry a significant morbidity and mortality profile if left untreated [25,26].

Struvite stones are mainly produced by supersaturation of magnesium ammonium phosphate and calcium phosphate. These stones are associated with urease-producing bacteria such as *Proteus mirabilis, Klebsiella pneumonia, Staphylococcus aureus, Pseudomonas aeruginosa, Providentia stuartii, Serratia, and Morganella morganii*. Urease mediates the pathophysiology of struvite stone formation by providing an alkaline environment that is critical for its formation, with urinary pH > 7.2 and elevated urine ammonia levels being predisposing conditions. Additionally, alkaline urine damages the protective layer of the urothelial surface, leading to the formation of bacterial biofilms that precipitate struvite stones [27–29].

## 3. Classes of urease inhibitors

Acetohydroxamic acid (AHA), an urease inhibitor, is the only FDA-approved drug. AHA is metabolized into CO_2_ and acetamide. CO_2_ is excreted with breath and accounts for 20–45% of the administered dose, while acetamide is eliminated in urine and contributes to 9–14% of the administered dose. The rest is excreted unchanged in the urine as AHA [30]. Putcha et al. demonstrated that AHA is rapidly absorbed from the gastrointestinal tract [30]. Another study revealed a direct relationship between AHA elimination and creatinine clearance, suggesting that patients with decreased renal function have lower excretion rates of this drug [31]. Feldman et al. demonstrated that in humans with normal renal function, the AHA half-life is 5–10 hours [32].

### 3.1. Class 1: metal-based urease inhibitors

Metal complexes, chelators, and nickel-substituting agents are the three subclasses of metal-based urease inhibitors.

#### 3.1.1. Metal-based complexes

Copper, zinc, cobalt, nickel, vanadium, silver, manganese, cadmium, iron, and gold are the most common metal complexes. Crucially, most of the copper metal complexes demonstrated greater urease inhibition among the assessed effective urease inhibitors, with IC_50_ values ranging from 0.46 μM to 41.1 μM. The IC_5__0_ values for copper, cobalt, and nickel Schiff base complexes have shown a promising effect; however, significant translational limitations have been shown. Most studies are constrained to *in vitro* enzymatic assays. Therefore, it will be more informative if future studies are conducted on intact bacterial cells, animal infection models, or human subjects to simulate the real microenvironment of bacterial growth. Moreover, copper complexes showed a promising effectiveness with very low IC_5__0_ = 0.46 µM; however, safety issues were not demonstrated in an *in vivo* model. The carcinogenicity of cobalt complexes might limit the use of these complexes against *H. pylori*. Collectively, clinical application of metal complexes as urease inhibitors requires further *in vivo* experimental approaches involving safety issues. Schiff base (–C = N–), an active pharmacophore, is an organic compound in which an imine or azomethine replaces the carbonyl group of an aldehyde or ketone. These compounds are widely utilized in industrial applications and are known for their diverse biological activities, including significant urease inhibition and antibacterial properties. Given the crucial role of transition metals, such as copper, nickel, cobalt, and zinc in various biochemical processes, metal complexes incorporating Schiff bases have been synthesized and widely investigated as potential urease inhibitors. Further highlighting of their potential in medicinal and pharmaceutical research is needed [2,33].

#### 3.1.2. Schiff base copper metal complexes

Many types of enzymes in biological systems depend on copper, an endogenous, biocompatible trace metal [34]. Accordingly, two azido-bridged Schiff base copper complexes, 1 and 2, were synthesized that demonstrated activities against urease (IC50 = 27.82 ± 0.35 μM) and complex 2 (IC_50_ = 25.23 ± 0.70 μM), respectively [2].

#### 3.1.3. Schiff base nickel metal complexes

The nickel (II) ion complexes have also shown antibacterial activity (39). Five Schiff base ligand-based complexes showed urease inhibition (IC_50_ = 63.00 μM) [2].

#### 3.1.4. Schiff base zinc metal complexes

Schiff bases and ZnBr2 exhibited lower inhibition rates when compared to these 24 and 25 compounds [2].

#### 3.1.5. Schiff base cobalt metal complexes

Cobalt complexes have shown a potent anti-urease activity. One complex demonstrated efficient activity against urease (IC_50_ = 0.35 ± 1.52 μM). Other metal complexes like vanadium, silver, manganese, cadmium, iron, and gold exhibit urease inhibition [2].

#### 3.1.6. Chelators

Chelators such as Hydroxamic acids, ethylenediaminetetraacetic acid (EDTA), and phosphoramidates inhibit urease by forming strong coordination bonds with the nickel ions at the enzyme’s active site. Each inhibitor bridges the two paramagnetic nickel ions, positioning them in an octahedral arrangement alongside the surrounding amino acid residues. This orientation closely resembles that of the enzyme’s natural substrate, effectively blocking its catalytic function [35].

#### 3.1.7. Hydroxamic acids (HXA)

**Figure 4.**
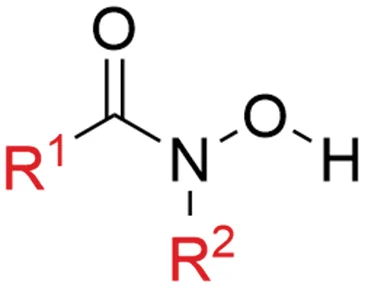
General chemical structure of hydroxamic acids, characterized by a carbonyl group bonded to a nitrogen atom that is further substituted with a hydroxyl group (-N–OH) and an additional substituent (R′). Created in ChemDraw19 (Version 19.0.1.28. Al-Zoubi, RM. (2025).

AHA, a derivative of hydroxamic acid [R-CONH-OH, R-C (OH)=NOH] (HXA) with a terminal O = C-NHOH functionality, is a significant class of urease inhibitors that was identified by Kobashi et al. in 1962, as effective metal chelates (Figure 4).

Hydroxamic acids attach themselves to the metal ions in the enzyme’s active site. AHA is a synthetic lead molecule that is stable, slightly acidic, and readily soluble in water. It shares structural similarities with urea [36,37]. A study demonstrated that animals’ *H. pylori*-induced gastritis was reduced in a dose-dependent way with the administration of AHA at doses of 100, 500, and 2500 ppm over the course of the trial. Animals given a 2500 ppm AHA following an *H. pylori* infection likewise showed suppression of stomach ulcers [35].

#### 3.1.8. Phosphoramidates

Phosphorodiamidates (PPDs) are strong inhibitors of soil bacterial urease, including N-acyl derivatives of phosphoric triamides and amido derivatives of phosphoric and triphosphoric acids. The ureases produced from the entire cells or cell extracts of *Morganella* morganii, *Mycobacterium smegmatis, Proteus vulgaris, Providencia rettegeri*, and *Ureaplasma urealyticum* demonstrated significant inhibition of urease. The hydrolytic conversion of PPD to diaminophosphate (NH2)_2_PO_4_ (DAP) explains the PPD’s compatibility in the urease active site [38].

#### 3.1.9. Ethylenediaminetetraacetic acid (EDTA)

Nickel ions are crucial for the active site activity. These nickel ions can be sequestered by EDTA, which inactivates the urease enzyme. Moreover, EDTA can inhibit the synthesis of urease in certain organisms. For instance, studies on two strains of *Cryptococcus neoformans* showed that EDTA more strongly suppressed urease synthesis in *C. neoformans var. gattii (Cryptococcus gattii) than in var. Neoformans (Cryptococcus neoformans)* [39].

#### 3.1.10. Nickel-substituting agent

Nickel is essential for urease activity due to its unique properties, which also ensure the correct orientation and activation of the substrate. Replacing nickel with Fe_2_+, Co_2_+, Mn_2_+, Zn_2_+, or Cu_2_+ significantly lowers the enzyme’s efficiency. The enzyme’s high catalytic rate and structural stability are enabled by nickel’s unique water-exchange rate, nucleophilicity, and ability to facilitate proton transfer [2].

### 3.2. Class 2: organic compounds

3.2.1.

Thioureas, Benzimidazole derivatives, Fluoroquinolones derivatives, Sulfonamides and sulfones, and Urea derivatives belong to the organic compound class

#### 3.2.2. Thioureas

This subclass includes phenylthiourea and fluorinated derivatives. The chemical formula for thiourea, an organosulfur compound, is SC(NH_2_). Its structure is comparable to that of urea (H_2_N-C(=O)-NH_2_), except that a sulfur atom replaces the oxygen atom. A class of chemicals with the formula (R1R2N)(R3R4N)C=S is referred to as “thiourea.” A study demonstrated numerous advantages, including antibacterial [40]. Thiourea derivatives with more inhibitory power than their urea counterparts have been created in recent years as a result of numerous studies looking into thiourea-based urease inhibitors [41]. Accordingly, compounds with substituents that contain lone electron pairs and those with functional groups affixed to the phenyl or heterocyclic ring around the thiourea core significantly impact the urease inhibitory action (IC_50_ = 8.43 µM). A direct comparison of the degree of inhibition attained by the monosubstituted N-benzoyl thiourea and thiourea demonstrated the advantageous effect of the benzoyl group on the inhibitory activity toward *Canavalia ensiformis (C. ensiformis)* urease (IC_50_ = 5.53 ± 0.02 to 91.50 ± 0.08 μM), *in vitro*, compared to conventional thiourea (IC_5__0_ = 21.00 ± 0.11 μM) [42]. Many N,N’-disubstituted thioureas have been identified as urease inhibitors. It is possible that N-monosubstituted thiourea with a small thiourea motif could bind as thiourea into the active pocket. According to the findings, N-monosubstituted aroylthioureas bind to the urease active site as predicted [43].

#### 3.2.3. Benzimidazole derivatives

Benzimidazole is a heterocyclic aromatic organic molecule resulting from the fusion of a benzene ring and an imidazole ring; this fusion produces a bicyclic structure with the molecular formula C_7_H_6_N_2_. Benzimidazole derivatives inhibit urease by interacting with the enzyme’s active site, often through coordination with its nickel ions [44,45]. The benzimidazole derivatives exhibited high urease inhibition activity (IC_50_: 5.85–20.82 µM) [46,47]. Moreover, the enzyme activity of purified *H. pylori* urease was found to be dose-dependently inhibited by omeprazole. Omeprazole equally reduced the growth of both mutant urease-negative and urease-positive *H. pylori* strains *in vitro* [48].

#### 3.2.4. Fluoroquinolone derivatives

Ciprofloxacin is a second-generation fluoroquinolone antibiotic used to treat bacterial infections. Its main mode of action is to inhibit bacterial DNA gyrase and topoisomerase IV. Quinolones have shown urease inhibitory activity (IC_50_ of 58.92 µM) [49].

#### 3.2.5. Sulfonamides and sulfones

The sulfonamide group (-SO_2_NH_2_) is present in a class of synthetic antibacterial agents known as sulfonamides. As anti-bacterial agents, their action involves blocking the dihydropteroate synthase enzyme, a key component of the folic acid synthesis pathway, which is essential for bacterial growth. Thiophene-tagged sulfonamides were recently reported in a study as urease inhibitors at a concentration of 15 µg/mL. Pyrazoletriazine-based sulfonamides were shown to be dual and powerful inhibitors of urease and tyrosinase, and their synthesized derivatives demonstrated greater promise than conventional thioureas. Substituted sulfonyl chlorides were used to create a limited library of chemicals based on medication derivatives. Interestingly, all of the drug variants had better effectiveness against the urease enzyme than the industry standard, thiourea [50,51]. Sulfathiazole is reported to be the closest structurally to thiophene-tagged sulfonamide, which possesses anti-urease properties where 70% of the drug is excreted unchanged in urine, and 17% is acetylated and excreted as N-acetylsulfathiozole [52].

### 3.3. Class 3: natural products

Plant-derived natural products have received significant attention as potential sources of urease inhibitors due to their antibacterial and anti-inflammatory properties [53]. Plant-derived compounds such as flavonoids, tannins, and alkaloids have shown promising outcomes on urease-related diseases. The mechanism of urease inhibition involves the chelation of nickel ions at the enzyme’s active site, thereby disrupting its activity [54,55]. For example, garlic extracts, cinnamon, cranberry juice, *Pteleopsis suberosa*, and black myrobalan, have different levels of effectiveness. *Radix Scutellariae, Radix Isatidis*, and *Rhizoma Coptidis* demonstrated a strong efficacy against *H. pylori* with MIC ≤ 7.8 mg/mL. The efficacy of plants such as *Rhizoma rhei, Flos Lonicerae Japonicae*, and *Cortex phellodendri* demonstrated MIC < 15.6 mg/mL to ≤62.5 mg/mL. The literature reviewed suggests that the longstanding gastrointestinal use of these plants for treating chronic gastritis and associated peptic ulcers lends credence to their efficacy and safety [56]. This combination of antimicrobial and urease-inhibiting properties positions these herbs as promising alternatives for treating *H. pylori* infections.

#### 3.3.1. Flavonoids

Flavonoids are a category of plant-derived chemicals with various bioactivities due to their polyphenolic structure. More than 9,000 structures of flavonoids have been identified and categorized based on their structural scaffolds (C6-C3-C6 skeleton). Structural variations in hydroxylation, methoxylation, and glycosylation influence their biological activities, including urease inhibition [57]. Genistein, an isoflavone widely produced by plants of the Fabaceae family, was found to inhibit *H. pylori* urease by 50%. While catechins, quercetin, and naringenin weakly inhibited the growth of *H. pylori* [54,58].

#### 3.3.2. Berberine

A natural substance is a principal component of *Coptis chinensis* and *Phellodendron amurense*, utilized to treat diarrhea and other gastrointestinal disorders. It exhibits considerable anti-urease activity against *H. pylori*. Among the berberine derivatives studied, 8-butyl berberine exhibited the most potent urease inhibitory activity, with 73.9% inhibition at a concentration of 1 mg/mL. This makes it a promising candidate for anti-*H. pylori* therapy, showing both bacterial growth suppression and urease inhibition [59].

#### 3.3.3. Ursolic acid

A pentacyclic triterpenoid in many plants (particularly *Mume Fructus*) inhibits urease and can potentially cure *H. pylori*-induced gastrointestinal problems [60]. Ursolic acid directly inhibits *H. pylori*‘s urease enzyme by blocking the proton pump (H+/K+-ATPase), lowering gastric acid production in the end phase, and increasing prostaglandin E2 (PGE2) levels. Ursolic acid destabilizes bacterial cell integrity. In *H. pylori* cells, studies have shown that nuclear deformation, chromatin condensation, and enhanced apoptosis are concentration-dependent processes [61].

#### 3.3.4. Sulforaphane (from cruciferous vegetables)

Isothiocyanate compound demonstrates potential urease inhibition; however, sulforaphane does not directly eradicate *H. pylori*. It exhibits indirect protective effects by reducing oxidative stress and lipid peroxidation in the gastric mucosa. This is achieved through increased glutathione (GSH) levels and decreased malondialdehyde (MDA) concentrations; sulforaphane’s cytoprotective and antioxidative properties suggest a complementary role in managing *H. pylori*-associated oxidative stress and inflammation [62]. Probiotic bacteria can inhibit *H. pylori* via both immunological and non-immunological processes. Lactobacillus can suppress *H. pylori* growth, block the action of its urease, and reduce *H. pylori* adhesion to cell lines, making them useful in preventing *H. pylori* colonization of the stomach [55]. Furthermore, probiotic Lactobacillus strains exhibit antimicrobial activity against the proliferation and urease activity of *Proteus spp*., thereby eliminating stone formation caused by these bacteria [63].

#### 3.3.5. Palmatine (Pal)

A natural sulfhydryl-targeting urease inhibitor derived from *Coptis chinensis* has been shown to effectively inhibit the growth of *H. pylori* under both neutral and acidic conditions (MIC = 100 to 200 µg/mL). Pal effectively inhibited *H. pylori* urease (HPU) and JBU in a dose-dependent manner (IC_5__0_ = 0.53 ± 0.01 mM) for HPU and (IC_5__0_ = 0.03 ± 0.00 mM) for JBU. Molecular docking experiments showed that Pal interacts with sulfhydryl groups but does not bind to the active site Ni^2 +^ metal ion [64]. These findings suggest that Pal could be a candidate for developing novel urease inhibitors with potential therapeutic applications for treating *H. pylori* infections and related gastrointestinal diseases. Collectively, the natural products’ potency as urease inhibitors needs further studies due to the heterogeneous outcomes. Moreover, different studies used different sources for the urease enzyme, such as jack bean urease. In addition, the stability, bioavailability, and biological kinetics of the natural product components, such as polyphenols, are not fully studied in the mentioned literature. Until these compounds are subjected to rigorous pharmacokinetic characterization and standardized *in vivo* models, their designation as “promising candidates” should be interpreted cautiously.

### 3.4. Class 4: peptide-based inhibitors

Peptides typically consist of between 2 and 50 amino acids, making them smaller than proteins [65]. Studies have shown that amphiphilic peptides have been explored as potential urease inhibitors, particularly due to their relevance in treating *H. pylori* infections. The peptides exert their inhibitory effect by binding to the enzyme’s active site through the chelation of nickel ions (Ni^2 +^), which are essential for urease activity. Since *H. pylori* urease also depends on nickel ions for its function, these peptides have the potential to inhibit it as well. *In vitro* experiments demonstrated strong urease inhibition (IC_5__0_ = 20.5 ± 0.01) [66,67]. Moreover, a study by Houimel et al. found that peptides that bind specifically to *H. pylori* urease were selected from libraries of random peptides displayed on lamentous phages in the context of pill coat protein, two peptides that can bind and can inhibit *H. pylori* were identified by screening of a highly diverse 25-mer combinational library and two newly constructed random 6-mer peptide libraries on solid-phase *H. pylori* urease holoenzyme. Both peptides were chemically synthesized, and their inhibition constants were determined to be 47 μM and 30 μM for the 24-mer and 6-mer peptides, respectively. Both peptides showed a specific inhibitory effect on *H. pylori* urease but not *Bacillus pasteurii* urease [68].

### 3.5. Class 5: hybrid molecule inhibitors

Hybrid molecules are compounds made to combine the advantages of two or more urease inhibitor classes. This strategy seeks to enhance their pharmacokinetic characteristics, efficacy, and selectivity. Hybrid compounds that combine two or more pharmacologically significant structures can exhibit higher biological activity against *H. pylori* infections, which are linked to gastric ulcers and cancers, as well as managing urease-related kidney stones and urinary tract infections (UTIs). They have used this method to develop urease inhibitors, concentrating on maximizing their inhibitory capabilities and overall therapeutic potential, in keeping with earlier studies on developing enzyme inhibitors, such as α-amylase and α-glucosidase inhibitors [69]. There are no licensed urease inhibition medications in human medicine that are explicitly formulated as metal-organic frameworks (MOFs) or synthetic hybrid molecules. Due to challenges related to human toxicity, biocompatibility, target specificity, and stability in biological systems without premature degradation, research, and development have advanced significantly, particularly in the design of medications and preclinical investigations.

#### 3.5.1. Metal-organic salts

The organic component of metal-organic salts (MOSs) enhances the accuracy of inhibition, as metals interact with the nickel ions in the urease’s active site. MOSs depend on non-covalent interactions between metal ions and organic cations, as opposed to conventional coordination complexes, which are bound together by covalent bonds. These compounds have garnered more attention in recent years due to their intriguing potential for a wide range of applications and distinctive architectures [70].

#### 3.5.2. Polymer-based and dual-targeting hybrids molecules

Experimental polymers enhance the inhibition of bacterial enzymes, improve stability, increase bioavailability, and prolong the activity of these enzymes. Chitosan nanoparticles serve as a delivery vehicle, improving the stability and controlled release of AHA, while also demonstrating antimicrobial properties [71]. The recently created hybrid compounds, which are produced by combining ciprofloxacin or norfloxacin with derivatives of thiouracil and thioxopyrimidine, have a dual action that targets both urease inhibition and antibacterial activity. The urease inhibitory activity shows IC_50_ values ranging from 9.71 to 80.64 μM [72]. The urease inhibition demonstrated by these hybrids was substantially more powerful than the individual components, like ciprofloxacin or norfloxacin, and thiourea, demonstrating the synergistic effects of combining these drugs.

### 3.6. Class 6: mechanism-based inactivators (suicide inhibitors)

A suicidal inhibitor irreversibly inactivates (or mechanism-based inhibitor), binds to the target enzyme, and undergoes a catalytic reaction.

#### 3.6.1. Boronic acids and Boronates:

Boric and boronic acids have been investigated as urease inhibitors.Boric acid inhibits urease by building complexes with the enzyme’s active site through interactions with nickel ions (Ni(II)) in the active site *in silico* [73]. This competitive inhibitory process is comparable to the binding structure of urea. Additionally, they demonstrate effectiveness against various urease enzymes, including those from Klebsiella aerogenes, soybean, pigeonpea, and Proteus mirabilis. Additionally, the possibly synergistic effect between boric acid and the nitrification inhibitor 3,4-dimethylpyrazole phosphate (DMPP) was examined. The findings showed that boric acid improved the nitrification inhibition efficiency of DMPP and reduced ammonia volatilization. This demonstrates how boric and boronic acids can function as inorganic urease inhibitors, similar to tavaborole (AN2690). Research by Benini et al. suggested that B(OH)_3_ interacts with Ni(II), proposing a mechanism where boric acid mimics urea in binding topology, acting as a competitive inhibitor [74].

#### 3.6.2. Phosphoramide derivatives

Phosphorodiamide compounds, known for their urease-inhibiting properties, have been primarily explored in agricultural contexts to enhance nitrogen use efficiency in fertilizers. Their application in human medicine, particularly for treating urease-related conditions, remains under investigation. Many phosphoramidates and related derivatives have been studied for their urease-inhibiting activities in whole-cell systems.

#### 3.6.3. Bis (N-methylaminomethyl)phosphinic acid

*H. pylori* and other bacteria’s urease were effectively inhibited by bis(N-methylaminomethyl) phosphinic acid. A suicide substrate, N-(n-butyl) thiophosphoric triamide (NBPT), was first used in agriculture in 2017. The enzyme hydrolyzes NBPT to produce monoamidothiophosphoric acid (MATP). MATP stays attached to the nickel ions in the active site, thereby rendering the enzyme inactive [75].

#### 3.6.4. Phenylphosphorodiamidate (PPD), N-n-butylthiophosphorictriamide (NBPT), and N-n-butylphosphorictriamide (NBPTO)

Molecular modeling studies demonstrated the importance of properties like the degree of conformational freedom, the volume of the fragment involved in the enzyme interaction [76].

#### 3.6.5. Uroofamide (N-(diaminophosphinyl)-4-uorobenzenamide)

Fluorofamide demonstrated potent *in vitro* urease inhibition, preventing ammonia release. However, its therapeutic potential was limited by significant acid instability, with a half-life (T_1_/_2_) of only 5.7 min at pH 2. *In vivo* studies of ferrets naturally infected with *H. mustelae* showed that while a single oral dose (50 mg/kg, per oral dose) could completely suppress bacterial urease activity, a repeat dosing regimen (50 mg/kg, three times daily) failed to eradicate the infection. This contrasted with the standard triple therapy (amoxicillin, metronidazole, and bismuth subcitrate), which achieved an 80% eradication rate. Histological analysis did confirm that fluorofamide treatment alone led to a profound reduction in bacterial numbers. A subsequent combination therapy of fluorofamide (50 mg/kg) with amoxicillin (10 mg/kg) administered twice daily for 7 days also failed to achieve complete eradication, though a reduction in bacterial load was observed in three out of five ferrets 21 days post-treatment. The study concluded that while fluorofamide could suppress urease and reduce bacterial load, urease inhibitors, either alone or in combination with antibiotics, offered limited therapeutic potential for complete eradication of Helicobacter infections in this model. This is likely because, *in vivo*, some bacterial populations do not rely solely on urease for survival. Nonetheless, the authors noted that the acid instability of fluorofamide was a key limitation, and the development of more stable urease inhibitors might hold future promise [77].

### 3.7. Class 7: noncompetitive inhibitors

Noncompetitive inhibitors bind to allosteric sites, altering the enzyme’s function without directly competing with urea at the active site.

#### 3.7.1. Polyphenol-based Inhibitors

Resveratrol is an example found in peanuts, berries, and grapes. Resveratrol-inhibited urease activity by 90% in three *H. pylori* strains, compared to 73% inhibition by AHA [78]. Resveratrol’s strong urease inhibitory activity suggests it could be a promising natural compound for combating *H. pylori*-related infections.

#### 3.7.2. Synthetic allosteric modulators

##### 3.7.2.1. Benzimidazole-based inhibitors

The efficacy of benzimidazole derivatives as urease inhibitors is closely related to their chemical structure and ability to interact with the urease enzyme, which is essential for H. pylori survival and virulence in the acidic gastrointestinal environment. Benzimidazoles often exhibit allosteric inhibitory action by binding to sites other than the enzyme’s active site, thereby altering its structure and function. This inhibition is critical in developing resistance-reducing therapies for *H. pylori* infections. Several benzimidazole compounds have been identified as effective urease inhibitors, showing substantial promise for targeting *H. pylori*. Examples include (2’−pyridyl) Benzimidazole Derivatives. The compounds inhibited the increase significantly, with one derivative (compound 507) having an IC_50_ value of 19.22 μM. This proved to be more effective than AHA (IC_50_ = 42.00 μM) [79].

### 3.8. Class 8: emerging strategies

#### 3.8.1. Nanomedicine

Nanotechnology-based strategies for enhancing the stability, activity, and reusability of enzymes are presented. According to the FDA and IUPAC, ‘nano’ refers to materials with a size range of 1–100 nm and, as a result, offer a high surface-to-volume ratio [80]. These advanced materials provide properties different from the bulk form, allowing them to penetrate biofilms and mucosal barriers; targeting *H. pylori* directly can also be functionalized with other drugs or compounds to boost their inhibitory and antibacterial activities.

##### 3.8.1.1. Silver nanoparticles (AgNPs)

The aforementioned nanoparticles demonstrate potential biocidal effects by conjugating to bacterial cell membranes, inhibiting cellular respiration, degrading key respiratory enzymes, inducing cell wall rupture, and increasing membrane permeability via pore formation, leading to cell death. As far as *H. pylori is concerned*, an adaptable and flexible urease-producing pathogen with a remarkable ability to colonize the stomach, AgNPs can be viewed as complementary alternatives for therapy and prophylaxis [81]. Studies show that conjugation of AgNPs with omeprazole enhances urease inhibition, antioxidant potential, and antibacterial activity [82].

##### 3.8.1.2. Zinc oxide nanoparticles (ZnO-NPs)

Functionalized ZnO-NPs (e.g., polyethyleneimine-coated ZnO) release Zn^2+^ ions, which inhibit urease activity by competing with nickel ions at the urease active site, thereby interfering with its enzymatic function. This disruption of urease function prevents *H. pylori* from effectively neutralizing stomach acid, thereby inhibiting it and leading to its eventual eradication. ZnO-NPs also disrupt bacterial membranes, contributing to their antibacterial effects. Their biocompatibility makes them a promising candidate for medical applications [83].

##### 3.8.1.3. Gold nanoparticles (AuNps)

Gold nanoparticles interfere with the structural integrity of urease by binding to its active site. It is often used in combination with antibiotics to improve antibacterial efficacy [84]. Nanoparticle stability and toxicity effects need to be addressed before the clinical trials. For instance, Ag-NPs have shown cytotoxicity to mammalian cells. Therefore, NPs’ applications as urease inhibitors need to be validated in animal models before human application.

#### 3.8.2. Gene therapy

Advanced experimental methods could be developed to mute the urease gene in *H. pylori*, making the bacterium less likely to survive in the stomach. The gene silencing strategy employs a genetic system (tetracycline-inducible) that allows for controlled expression of the urease gene in *H. pylori*. Silencing the gene inhibits the development of the urease enzyme, thereby eliminating its activity [36].

##### 3.8.2.1. siRNA-Based gene silencing

Small interfering RNAs (siRNAs) are designed to specifically target and degrade the mRNA of genes encoding key virulence factors in *H. pylori*, such as ureB (a subunit of urease) and cagA (associated with gastric cancer development). The study used small interfering RNAs (siRNAs) to investigate the expression and function of ureB and cagA. The SS1 strain of *H. pylori* was deemed a host for natural transformation. The siRNA targeting the ureB and cagA genes was introduced into the pGPU6/GFP/Neo siRNA plasmid vector for evaluation utilizing phenotypic and genotypic techniques. The inhibition rates of ureB and cagA gene expression were determined using qPCR. The expression levels of siRNA-ureB and siRNA-cagA in the recombinant strain SS1 were lowered by approximately 5000 and 1000 times, respectively, when compared to the native *H. pylori* strain. An early *in vitro* study of siRNA-ureB revealed suppression of urea enzyme activity. These studies imply that siRNA could be a significant new tool for gene silencing *in vitro* and the development of RNAi-based anti-*H. pylori* therapies [37].

### 3.9. Novel synthetic compounds

Based on their mechanisms and structural interactions, they can be categorized into multiple subgroups.

#### 3.9.1. Disulfides (N-Arylacetamide disulfides)

To develop novel urease inhibitors, 38 N-arylacetamide disulfides were synthesized. Some synthesized compounds showed high inhibitory effectiveness against urease in both cell-free and intact cells, with negligible cytotoxicity to mammalian cells at concentrations up to 250 μM. 2,2’-dithiobis (N-(2-uorophenyl)acetamide) (d7), 2,2’-dithiobis(N-(3,5-diuorophenyl)acetamide) (d24), and 2,2’-dithiobis(N-(3-uorophenyl)acetamide) (d8) were identified as the most active inhibitors with IC_50_ of 0.074 ± 0.008, 0.44 ± 0.04, and 0.81 ± 0.02 μM, showing 32- to 355-fold higher potency than the positive control AHA. These disulfides have been shown to bind urease without covalently altering the cysteine residue and to block urease reversibly via a mixed inhibition mechanism [85]. These compounds could be effective since *H. pylori* urease shares structural similarities with JBU (commonly used in inhibition studies).

#### 3.9.2. Sulfonates & sulfamates (Imidazo[2,1-b] thiazole derivatives)

A series of compounds based on an imidazo[2,1-b]thiazole scaffold was developed. Among them, the most potent inhibitor was a compound identified as 2c. It exhibited strong urease inhibitory activity, with an IC_5__0_ of 2.94 ± 0.05 µM, approximately eight times more potent than the standard inhibitor thiourea (IC_5__0_ = 22.3 ± 0.031 µM). Enzyme kinetics studies revealed that compound 2c acts as a competitive inhibitor, meaning it directly blocks the enzyme’s active site. This mechanism is supported by molecular modeling, which showed that 2c forms multiple interactions with key amino acid residues at the active site of urease [86]. This targeted action is particularly relevant against *H. pylori*, as its urease possesses a well-defined active site containing two nickel ions, which are crucial for the enzyme’s function and are a common target for competitive inhibitors like 2c.

#### 3.9.3. Thiosemicarbazones

We propose a new family of N4-substituted thiosemicarbazones 3a–v as possible urease inhibitors. These novel N4-substituted thiosemicarbazones 3a-v, derived from distant chemical scaffolds, were investigated using advanced spectroscopic techniques, including FTIR, ^1^H NMR, ^13^C NMR, and ESI-MS, as well as single-crystal X-ray analysis for compound 3 g. The substances were tested for urease inhibitory potential. All newly synthesized compounds demonstrated considerable urease inhibition, with IC_50_ values ranging from 2.7 ± 0.320 to 109.2 ± 3.217 μM [87]. These covalent interactions with cysteine residues in urease make them potentially irreversible inhibitors.

#### 3.9.4. Benzimidazoles

They are aromatic heterocyclic organic compounds having a benzene ring fused with an imidazole ring. The imidazole ring is a component of many natural products, including purine, histamine, histidine, and nucleic acids. Due to its polar and ionizable ability, it proves to be characteristic of pharmacokinetics for lead molecules by enhancing their solubility [88]. Rarely studied for urease inhibition, benzimidazoles can form interactions with nickel ions or amino acid residues, leading to strong inhibition.

#### 3.9.5. Bi-heterocyclic Bi-amides (thiazole-oxadiazole derivatives)

N-(4-[(5-sulfanyl-1,3,4-oxadiazol-2-yl) methyl]-1,3-thiazol-2-yl) benzamide compounds showed potent inhibition (compound 9j, IC_5__0_ = 2.58 ± 0.02 μM, stronger than thiourea) [89]. They could be effective if they bind similarly to the *H. pylori* urease active site.

#### 3.9.6. UreG inhibitors

UreG is a helper protein crucial for enabling urease to function in bacteria such as *H. pylori*. Its main job is to deliver nickel, a necessary metal, into the urease enzyme to activate it, a process called maturation. The strategy of UreG inhibitors is to stop this helper protein from working. Researchers have found small molecules that block UreG’s function. When UreG is blocked, nickel cannot be inserted, and the urease enzyme remains inactive. This approach has been shown to stop bacterial growth in lab tests effectively [90]. Because UreG is essential for activating urease but is not the main enzyme itself, it represents a promising new target for developing drugs that could bypass some common resistance mechanisms.

## 4. Urease inhibitors and FDA approval

In the clinical setting, only one direct urease inhibitor has received FDA approval. The only urease inhibitor is AHA, which was approved in 1983 as Lithostat for use as an adjunctive medicine in the chronic, refractory type of urinary tract infections caused by urease-producing bacteria like Proteus mirabilis, especially to prevent struvite stone formation [91]. Although it is effective, there are side effects and specific indications that limit its use [31]. An indirect approval, Bismuth salts (subsalicylate or subcitrate), which are used in *H. pylori* therapy. They disable urease by binding metals and hindering the maturation of enzymes, rather than through the active site [92]. Disulfiram, however, is an FDA-approved drug for purposes other than a urease inhibitor – alcohol dependence treatment under the trade name Antabuse. Its anti-urease action is off-target and considered experimental [93]. Evidence supports the notion that disulfiram and its metabolites can disrupt urease (through cysteine interaction), but there is no approval for any clinical consideration targeting urease. Therefore, AHA is the only one available with direct counteractions on the urease enzyme in medical practice (Table 1). Despite the promising potency of eight mechanistic classes of urease inhibitors, acetohydroxamic acid remains the sole FDA-approved urease inhibitor, highlighting a persistent translational gap. Many potent inhibitors did not succeed in transitioning from *in vitro* activity to clinical utility, “promising candidate for future investigation.” Importantly, the clinical application of the newly discovered urease inhibitors requires pharmacokinetics, selectivity, stability, or bactericidal activity in an animal model. Therefore, *in vivo* efficacy in standardized infection models is an obligation before new compounds are designated as clinically promising. Detailed subclass classification, mechanism, and experimental evidence are provided in Supplementary Table S1.

**Table 1.** Overview of urease inhibitor classes, mechanisms of action, and levels of experimental evidence.

Class	General mechanism	Level of evidence
Class 1: metal-based urease inhibitors	Nickel coordination / chelation	*In vitro*, *in vivo*, clinical
Class 2: organic compounds	Active site interaction / structural modification	Mostly *in vitro*
Class 3: natural products	Nickel chelation / bioactive modulation	*In vitro*
Class 4: Peptide-based inhibitors	Enzyme binding / nickel chelation	*In vitro*
Class 5: hybrid molecule inhibitors	Dual-targeting mechanisms	*In vitro*
Class 6: mechanism-based (suicide) inhibitors	Irreversible enzyme inactivation	Experimental
Class 7: noncompetitive inhibitors	Allosteric modulation	*In vitro*
Class 8: emerging strategies	Nanotechnology / gene-based approaches	*In vitro*

### 4.1. Pharmacological profile of acetohydroxamic acid

AHA is the only FDA-approved drug and is the only drug that has been tested in human trials. AHA is metabolized into CO_2_ and acetamide. CO_2_ is excreted with breath and accounts for 20–45% of the administered dose, while acetamide is eliminated in urine and contributes to 9–14% of the administered dose. The remainder of the dose is excreted unchanged in the urine as AHA [30]. Putcha et al. conducted an experiment regarding AHA, which demonstrated that AHA is rapidly absorbed from the GI tract [30]. Further study of urinary excretion data revealed a direct relationship between AHA elimination and creatinine clearance, suggesting that patients with decreased renal function exhibit decreased excretion rates of this drug [94]. Feldman et al. examined the AHA pharmacological profile in rats and humans. It demonstrated that in humans with normal renal function, the AHA half-life is 5–10 hours. Half-life and percent of dose recovered in urine seem to be related to the dose and renal functions [95].

As urease inhibitors theoretically provide promising uses in treating different pathologies, there is a lack (or even absence in some agents) of experiments that have been conducted to assess the efficacy, reliability, and side effects of different urease inhibitors in human trials. Even in some human trials on urease inhibitors (AHA), many GI and neurological side effects have been reported, which may limit further human trials on AHA [94]. A lack of human trials may limit the clinical use and FDA approval of the different agents. We recommend doing further human trials on urease inhibitors. If these agents were clinically effective, they may play a significant clinical role in treating *H. pylori* infections or urinary tract infections, in conjunction with traditional regimens used to treat these infections or at least decrease the incidence of resistance development in urease-producing bacteria.

## 5. Therapeutic uses of urease inhibitors

### 5.1. Helicobacter pylori

*H. pylori* possesses many virulent factors that allow it to survive, such as extreme genome variation, motility, and urease, which is the enzyme we will focus on in this review. This pathogen needs to be eradicated, as it may cause gastric carcinoma and gastric MALT lymphoma [96]. Eradication of *H. pylori* results in rapid healing of active ulcers and low recurrence rates. Triple therapy, which consists of a proton pump inhibitor (PPI), clarithromycin, and amoxicillin (metronidazole if amoxicillin resistant), is considered the best therapy approach. On the other hand, quadruple therapy, consisting of bismuth subsalicylate, metronidazole, and a tetracycline with PPI, is considered in areas of clarithromycin resistance, with an eradication rate approaching 90% [97]. Urease is essential for *H. pylori* survival. Therefore, targeting it with urease inhibitor agents may help eradicate *H. pylori*. Many natural, semi-synthetic, and synthetic agents were developed. However, their use in clinical trials was limited due to side effects and toxicities [98]. Accordingly, Zhou et al. investigated the urease-blocking and bactericidal properties of Pal. Regarding urease blockades (IC_50_ = 0.53 ± 0.01 mM and 0.03 ± 0.00 mM) for HPU and JBU, respectively. AHA, a well-known urease inhibitor used as a positive agent, had IC_50_ values of 0.07 ± 0.01 mM for HPU and 0.02 ± 0.00 mM for JBU.

Pal showed inhibitory efficacy against *H. pylori* in both neutral and acidic environments, according to the results, with the inhibitory impact appearing to be more pronounced in the acidic environment than in the neutral one. A well-known antibiotic, metronidazole, was used as a positive control [64]. Moreover, Hanaa Shalaan reported that AHA significantly lowered urease activity in AHA-treated isolates compared to normal isolates, and almost full inhibition was already achieved at an inhibitor concentration of 2.5 mM. Baicalin is another agent that was experimented with in this study. It lowered urease activity at concentrations of 2 and 4 mM. However, urease activity in the isolates returned to normal after 20 minutes, indicating significant urease blocking activity of approximately 50% with 8 mM treated bacillacin. Moreover, Ebselen, another urease-blocking agent, was also tested in this study. It revealed significant urease-blocking properties; 0.003 mM was sufficient to achieve a 71% reduction compared to controls [99]. Similarly, another study investigated the efficacy of fluorofamide, a potent urease inhibitor, on the eradication of *H. pylori* infection. Histological samples showed a significant decrease in *H. mustelae*, fluorofamide (50 mg/kg per os, three times a day) failed to eradicate the infection compared to triple therapy, also a combination of amoxicillin and fluorofamide (seven days of treatment with 50 mg/kg fluorofamide plus 10 mg/kg amoxicillin per os twice a day) failed to eradicate the infection [77]. Matongo et al. investigated the use of honey fraction (HS) and manuka honey (HM) in urease inhibition, While the diethyl ether extract of HM demonstrated 45 and 51% inhibitory activities against urease from *H. pylori* (369C) and *H. pylori* (ATCC 43526), respectively, the chloroform extract of HS demonstrated 48 and 42% inhibitory activities against these two bacteria. According to the Dixon plot, the HM extract exhibited a heterogeneous inhibition pattern with reversible inhibition kinetics [100]. Hassan et al. investigated the effects of rabeprazole, omeprazole, and lansoprazole on *H. pylori* urease activity; all agents inhibited urease by binding to the enzyme’s active site, with rabeprazole approximately 10 times more potent than the others. Additionally, lansoprazole and omeprazole showed IC_5__0_ values against cellular urease that were 10 times higher than those against cell-free urease. However, the IC_5__0_ values of rabeprazole and lansoprazole against cellular urease didn’t differ from those against cell-free urease [98]. Goldie et al. investigated the inhibitory effect of AHA on urease, and the highest inhibition occurred at AHA concentrations of 200–1000 mg/L. However, inhibition was temporary, as the urease level was positive again after 24 hours. AHA didn’t affect the survival of the organism [101]. Accordingly, Phillips et al. assessed the bactericidal effect of AHA on eight isolates of *H. pylori*. AHA carried an inhibitory effect on eight isolates and a bactericidal effect on seven of these eight isolates. MIC ranged from 200 to 400 mg/L, and the MBC (minimum bactericidal concentration) ranged from 200 to 1600 mg/L. Also, AHA reduced MICs for tetracycline, metronidazole, and amoxicillin, and, for some isolates, reduced minimum bactericidal concentrations (MBCs) for these drugs; for a few isolates, MBCs increased for all four antimicrobial drugs. Moreover, Qiang Lu et al. investigated the inhibitory effect of Zanthoxylum Nitidium, a traditional Chinese medicine, on HPU and JBU. It showed higher enzyme activity on HPU (IC_50_ = 1.29 ± 42 0.10 mg/mL) compared to JBU (IC_50_ = 2.04 ± 0.27 mg/mL). It also revealed that the binding type of Z. Nitidum to HPU was slow-binding and mixed-type, whereas it inhibited JPU by a slow-binding and noncompetitive type [102]. Similarly, Qiang Lu also investigated the inhibitory effect of Sanguinarine, a natural alkaloid isolated from Z. nitidum (SNG), on *H. pylori* and JBU. It significantly suppressed the activity of both ureases in a concentration and time-dependent manner with IC_50_ of 0.48+-0.14 mM and 0.11+-0.02 mM, respectively, compared to AHA, a well-known urease inhibitor, (0.06 ± 0.01 mM for HPU and 0.03 ± 0.00 mM for JBU, respectively) [100]. Moreover, Hyun Jun Woo reported the inhibitory effect of Zerumbone, in which *H. pylori* was cultured and treated with either 0.5, 10, or 20 μM Zerumbone concentration; urease activity was decreased in a dose-dependent manner to 56%, 66%, and 73% of the untreated control [103]. Xiao-Dan Yu investigated the inhibitory effect of patacholi alcohol (PA) on *H. pylori* urease, comparing it with AHA as a reference drug, and reported that PA inhibited in a dose-dependent manner. *H. pylori* urease with values of 2.67 ± 0.79 in a noncompetitive inhibition fashion. In summary, PA’s targeted antibacterial effect on *H. pylori* and its ability to inhibit urease highlight its potential as a therapeutic agent for treating *H. pylori* infections [104].

### 5.2. Struvite stones

*Proteus mirabilis (P. mirabilis*) is a Gram-negative bacterium that has facultative anaerobic characteristics and a rod-shaped morphology, a member of the Enterobacterial order and the Enterobacteriaceae family. Patients with a Proteus infection typically manifest clinical symptoms consistent with urethritis, cystitis, prostatitis, or pyelonephritis. A medical record revealing recurrent nephrolithiasis could suggest a persistent Proteus infection, given that a history of stone formation may indicate a chronic Proteus infection. These bacteria produce urease, a bacterial enzyme essential to the crystallization process. The crystals of struvite and amorphous carbonate apatite are the most often occurring solid components of pathogenic urinary stones. There are two primary mechanisms involved in the production of these stones. The nucleation of solid phases is the first step, and the agglomeration of crystalline and amorphous precipitated phases is the second step. P. mirabilis-instigated UTIs and CAUTIs can also be exacerbated by the development of urolithiasis in the bladder and kidneys, potentially causing irreversible renal impairment [105]. Due to this wide range of complications, some studies have been conducted on the potential clinical implications of urease inhibitors, which may help prevent kidney infections and stone formation. The urease-negative mutant of P. mirabilis was studied in a catheterized mouse model. While this mutant successfully colonized the bladder, competitive urease effects directly prevented stone formation, confirming the role of urease in pathogenesis. This suggests that urease inhibitors could prevent stone formation and kidney infections but may not completely eradicate bladder colonization [106]. However, the strict dependence of struvite and carbonate apatite renal stones on the urease-producing germs confirms the need to combine bacterial urease inhibitor drugs with antibiotic treatment. Of the two anti-urease drugs, better results were obtained with AHA due to its minimal side effects and more powerful urease-inhibitor capacity. Thus, stone recurrence was avoided even in patients with urinary infections that did not respond to specific antibiotic treatment [107]. Therefore, a comprehensive treatment plan often involves a combination of antimicrobial therapy, urease inhibition, urinary acidification, and, when necessary, surgical intervention to remove existing stones.

#### 5.2.1. Urea derivatives (2-mercaptoacetamide(2-MA))

2-MA is a structural homologue of urea that exhibits competitive activity against urease. Prior research has investigated *P. mirabilis* virulence-associated enzymes as possible treatment targets. A library of N-alpha mercaptoamide dipeptide inhibitors targeting the Zap A protease, a virulence factor crucial to *P. mirabilis* pathogenesis, was previously created and examined in detail by Carson et al. [105].

2-MA has emerged as a promising urease inhibitor targeting *Proteus mirabilis*, a key pathogen in urinary tract infections and catheter encrustation. *In silico* and quantum-level studies show that 2-MA inhibits urease by mimicking urea or forming a tetrahedral intermediate, with the latter providing stronger suppression. Unlike AHA, which has higher urease affinity but significant cytotoxicity, 2-MA offers low toxicity, affordability, and dual activity-blocking urease and biofilm formation. Though effective at 10 mM, its clinical application via direct bladder washouts using a Foley catheter makes it suitable for weekly prophylaxis. Additionally, the presence of a thiol group enhances its binding potential. 2-MA provides a valuable platform for designing potent analogs and may serve as a safer alternative to AHA in treating *P. mirabilis*-related infections [108].

#### 5.2.2. Dimethyl sulfoxide (DMSO)

DMSO is structurally similar to urea, used as a solvent for urease inhibitors, and an effective treatment for interstitial cystitis/bladder pain syndrome (IC/BPS). Urease is highly sensitive to noncompetitive inhibition by DMSO (Ki approximately 6 mmol/L). DMSO inhibited urease activity in whole cells, limited bacterial growth in media containing urea, and slowed the increase in pH that occurred in artificial urine medium. This makes it a potential treatment for *P. mirabilis* [22]

#### 5.2.3. Acetohydroxamic acid

Propionohydroxamic acid (PHA) belongs to the class of hydroxamic acids, which are characterized by the functional group –C(=O)NHOH. The low dose (120 mg/day) for 1 week, followed by 60 mg/day, effectively binds to the nickel ions in the active site of the urease enzyme [109], showing excellent anti-urease activity with nearly normal absence of side effects, allowing long-term administration [6].

### 5.3. Probiotic lactic acid

#### 5.3.1. Lactobacillus

Lactobacillus strains that reside in the urinary system produce lactic acid, which interacts with the catalytic domain of urease to modulate its activity. Additionally, it demonstrates the capacity to inhibit the crystallization process, which is one of the initial phases of stone development in the urinary tract. The presented experiments demonstrated a multidirectional antagonistic impact, including urease inhibitory and antibacterial characteristics against *P. mirabilis*. The kinetic approach and *in silico* analysis were used to investigate the impact of lactic acid on urease activity. The crystallization process was impeded when specific strains of Lactobacillus were present. These results show that Lactobacillus and lactic acid have a great impact on the development of urinary stones, which in the future may help to support the treatment of this health problem [110].

### 5.4. Boric acid and boronic acids

The detailed mechanism of bacterial urease inhibition by boric acid and boronic acids is not established, but it likely involves the metallocentre. In analogy with serine hydrolases, the metal chelate is at the ligand position normally occupied by the substrate. They may hold potential pharmaceutical value in minimizing urinary stone formation and could provide useful probes for studying the active site of urease and other hydrolytic enzymes. Various coumarin derivatives have been synthesized, particularly Schiff bases formed by reacting 4-aminocoumarin with aromatic aldehydes. These compounds have been evaluated for their efficacy and cost-efficient treatments for preventing kidney stones and related complications from excessive urease activity [111]. Synthesized promising results *in vitro*. The urease inhibition by these derivatives is attributed to their ability to bind to the enzyme’s active site, thereby maintaining a lower urine pH, which is unfavorable for struvite stone formation. Some coumarin derivatives have undergone *in vivo* evaluation, demonstrating potential in preventing kidney stones and exhibiting anti-ulcer properties. These findings suggest a dual therapeutic benefit, addressing both urease-related stone formation and gastric ulcers [112].

*Klebsiella pneumoniae* is an urease-producing bacterium that is involved in kidney stone formation and urinary tract infections (*Klebsiella pneumoniae and Klebsiella oxytoca*-produced crystals, including Ca phosphate and Ca oxalate, respectively) [113]. Prevalence of multi-drug resistant (MDR) *K. pneumoniae* increases the mortality and morbidity, as well as the economic cost [114]. Accordingly, the combination of enhanced virulence and multidrug resistance of *K. pneumoniae* has the potential to utilize a non-antibiotic approach (urease inhibitors) to control Klebsiella-mediated urolithiasis.

### 5.5. Ethylenediaminetetraacetic acid (EDTA) and dimethylglyoxime (DMG)

EDTA and DMG reduce the nickel import and ureolytic activity of cells. In contrast, oxalic acid stimulated nickel import but reduced the ureolytic activity of cells. Additionally, 1,2,4-butanetricarboxylic acid strongly stimulated nickel import and slightly increased the ureolytic activity of cells [115].

### 5.6. Natural urease inhibitor extracts from *Emblica*

Natural urease inhibitor extracts from *Emblica officinalis* have demonstrated significant anti-urease activity against *K. pneumoniae*. Similarly, bioactive compounds derived from sumac fruit, pomegranate peel, and almond leaves have shown promising inhibitory effects on urease, offering alternative approaches to controlling urease-mediated infections [116].

### 5.7. Synergistic urease inhibition through combinations of tannic acid (TA), sodium fluoride (NaF), and AHA

These compounds significantly reduce ureolytic activity in *K. pneumoniae*, thereby delaying ammonia production, increasing the bacterial growth lag phase, and preventing pH elevation-key factors in UTI recurrence, stone formation, and biofilm development on catheters. Low-dose TA and NaF minimize side effects by reducing the required AHA dosage, making them a promising alternative for managing carbapenem-resistant *K. pneumoniae* and other urease-associated infections [116].

### 5.8. Hepatic encephalopathy

The pharmacological use of AHA is expected to be practical and relatively safe, as it has been tested in several research studies [107]. AHA decreases the blood ammonia level, and its therapeutic application is restricted. 2-octynohydroxamic acid is an octanhydroxamic (n-hydroxyoctanamide; caprylhydrxamic acid, a derivative of OHA, a hydroxamate-based urease inhibitor that is less cytotoxic and mutagenic, and it reduces the level of ammonia in experimental rodents. Furthermore, it was found to lower the cerebellar glutamine in rats [22].

## 6. Conclusion

Urease remains a critical virulence factor in multiple pathogenic conditions, including *H. pylori* infection, and its inhibition continues to attract significant research interest. Although a wide range of compounds have been studied, only AHA has reached clinical approval, highlighting the gap between laboratory promise and therapeutic approaches. The emergence of hybrid molecules, nanotechnology-based systems, and natural product–derived inhibitors offers new opportunities for safer and more effective urease-targeted therapies. However, challenges such as toxicity, poor stability, and insufficient human data must be addressed before these agents can be widely adopted in clinical practice. Expanding clinical trials and exploring combination regimens with existing antimicrobials could pave the way for urease inhibitors to become integral components in the management of *H. pylori* infection, urease-mediated urinary stones, and HE.

## 7. Future perspective

Over the next 5–10 years, the field of urease inhibition is expected to shift from predominantly *in vitro* exploration toward clinically translatable strategies. Advances in structure-based drug design and computational modeling will likely enable the development of more selective and potent inhibitors targeting the urease active site and its accessory proteins, such as UreG. In parallel, nanotechnology-based delivery systems are anticipated to improve drug stability, bioavailability, and targeted delivery, particularly in challenging environments such as the gastric mucosa and urinary tract. Combination therapies integrating urease inhibitors with antibiotics or anti-inflammatory agents may become increasingly important to overcome antimicrobial resistance, especially in *Helicobacter pylori* infections. Furthermore, emerging gene-silencing approaches, including small interfering RNAs, could offer novel avenues for suppressing urease expression at the molecular level. Despite these promising directions, key challenges remain, including toxicity, off-target effects, and limited clinical validation. Future research should prioritize well-designed in vivo studies and clinical trials to bridge the gap between experimental findings and therapeutic application, ultimately establishing urease inhibition as a viable and effective strategy in the management of urease-mediated diseases.

## Supplementary Material

Appendix.docx

Supplementary_Table_1_MAZ_R2.docx

## Article highlights

- Urease remains a key therapeutic target in H. pylori, struvite stones and hepatic encephalopathy.
- Acetohydroxamic acid is the only approved urease inhibitor, underscoring a major translational gap.
- Metal complexes, natural products and hybrid scaffolds show potent preclinical anti-urease activity.
- Future progress depends on safer inhibitors, improved delivery systems and robust in vivo/clinical validation.

## Writing assistance

No writing assistance was utilized in the production of this manuscript.
