# Supplementary material for: Urease inhibitors: current advances, therapeutic potentials, and clinical challenges
Source: Future Med Chem. 2026 Jun 15;18(14):1907–23. doi: 10.1080/17568919.2026.2684497 (PMC13557569; doi:10.1080/17568919.2026.2684497)
Supplement: Supplementary file 1 [file IFMC_A_2684497_SM8839.docx]

| **Table 1S.** Summary of urease inhibitors categorized by class, mechanism of action, and level of experimental validation. | | | | |
| --- | --- | --- | --- | --- |
| **Class** | **Sub-class** | **First author (Ref.No)** | **Mechanism of Action** | **Phase/Level (in vitro, vivo, clinical)** |
| **FDA approved** | | | | |
| **Class 1**: Metal-based urease inhibitors | Hydroxamic acid (AHA) | Rego, Y.F., et al [44]  Griffith, D.P. [34] | Chelation of nickel ions in the urease active site | *In vivo* (animal trial) [38] Clinical Use [34] |
| **Non-FDA** | | | | |
| **Class 1**: metal-based urease inhibitors | Metal-based complexes: | Yang, W., Z. Peng, and G. Wang, [100] | Coordination with nickel ions at the urease active site | *In vitro* |
|  | Schiff base copper metal complexes: | Yang, W., Z. Peng, and G. Wang,[100] |  | *In vitro* |
|  | Schiff base nickel metal complexes. | Yang, W., Z. Peng, and G. Wang,[100] |  | *In vitro* |
|  | Schiff base zinc metal complexes | Yang, W., Z. Peng, and G. Wang,[100] |  | *In vitro* |
|  | Schiff base Cobalt metal complexes | Yang, W., Z. Peng, and G. Wang,[100] |  | *In vitro* |
|  | Chelators | Ohta, T., et al[38] | Chelators inhibit urease by forming strong coordination bonds with the Nickel ions at the enzyme’s active site. | *In vitro* |
|  | Phosphoramidates | Amtul Z et al[41] | Hydrolytic conversion in the active site, binding to nickel ions | *In vitro* |
|  | Ethylenediaminetetraacetic acid (EDTA) | Kwon Chung et al [42] | Sequestration of nickel ions, inhibiting enzyme synthesis/activity | *In vitro* |
|  | Nickel-substituting agents | Yang, W., Z. Peng, and G. Wang,[100] | Substitution of the active site nickel (Ni²⁺) with other metals (e.g., Fe²⁺, Co²⁺, Zn²⁺, Cu²⁺), which cannot replicate the precise catalytic environment, drastically reduces enzyme efficiency. | *In vitro* / Experimental Approach |
| **Class 2**: Organic compounds | Thiouria | Song, W.-Q., et al. [46] | Interaction with the active site; beneficial effect of the benzoyl group. | *In vitro* |
|  | Benzimidazole derivatives: | Saeedian Moghadam, E [49] | Interaction with the enzyme's active site often involves coordination with nickel. | *In vitro* |
|  | Fluoroquinolone derivatives: | Abdel-Aal, M.A., et al. [52] | Functionalization at piperazinyl N-4 position enhances inhibition. | *In vitro* |
|  | Sulfonamides and Sulfones | Ovung, A. and J. Bhattacharyya [56] Channar, P.A., et al. [57] | Dual inhibition of urease and tyrosinase; binding to active site | *In vivo[58]* |
| **Class 3**: Natural product | Flavonoids | Modolo, L.V., et al., [60] | Chelation of nickel ion at the enzyme binding site of urease | *In vitro* |
|  | Berberine | Han, Y.-H. et al [65] | Anti-urease activity; bacterial growth suppression | *In vitro* |
|  | Ursolic acid: | Kim, S.W., et al [67] | Additionally, it blocks proton pump  (H+/K+-ATPase), lowering gastric acid production in the end phase, and increasing prostaglandin E2 (PGE2) levels. | *In vitro* |
|  | Sulfuraphane | Chang, Y.W., et al.[68] | Besides chelating nickel enzymes, it reduces oxidative stress and lipid peroxidation in the gastric mucosa. | *In vitro* |
|  | Palmatine (Pal) | Zhou, J.-T., et al.[70] | Interacts with sulfhydryl groups, inhibits urease dose-dependently, but does  not bind to the active site Ni²⁺ metal ion. | *In vitro* |
| **Class 4**: Peptide-based inhibitors | Amphiphilic Peptides | Shah, Z.A., et al. [73] | Chelation of nickel ions (Ni²⁺) at the enzyme’s active site | *In vitro* |
|  | Phage-displayed peptides | Houimel, M., et al [74] | Specific binding and inhibition of *H. pylori* urease holoenzyme | *In vitro* |
| **Class 5**: Hybrid Molecule Inhibitors | Metal-organic salts (MOSs): | Duan, W.-L., et al.[76] | Metal ions interact with nickel; organic component enhances accuracy | *In vitro* |
|  | Polymer-Based (Chitosan) | Chang, S.-H. et al [77] | Delivery vehicle for AHA; also has antimicrobial properties | *In vitro* |
|  | Dual-Targeting Hybrids Molecules (Ciprofloxacin hybrids) : | Abdel-Aziz, S.A., et al., [78] | Combines urease inhibition and antibacterial (DNA gyrase) activity | *In vitro* |
| **Class 6:** Mechanism-Based Inactivators (Suicide Inhibitors) | Boronic acid and boronate | Costa, N.E.M., et al. [79] | Mimics urea binding topology; competitive inhibition via Ni(II) interaction. | *In vitro* |
|  | Phosphoramide derivative (e.g., NBPT) | Kafarski, P. and M. Talma[80] | Suicide substrate; hydrolyzed product binds irreversibly to nickel ions | Experimental (Agricultural) |
|  | Bis (N-methylaminomethyl) | Kafarski, P. and M. Talma [80] | Acts as a suicide substrate; effectively inhibits *H. pylori* and other bacterial ureases. | *In vitro* |
|  | Phenylphosphorodiamidate (PPD), N-n-butylthiophosphorictriamide (NBPT),  and N-n-butylphosphorictriamide (NBPTO). | Font, M.a., et al., [81] | Molecular properties (conformational freedom, HOMO orbitals, etc.) govern urease inhibitor activity. | *In vitro* / In silico (Modeling) |
|  | Uroofamide (N-(diaminophosphinyl)-4-uorobenzenamide). | Pope, A.J., et al. [82] | Potent urease inhibitor; limited by acid instability | *In vitro* & *In vivo* (animal). |
| **Class 7:** Noncompetitive Inhibitors | Polyphenol-Based Inhibitors: | Paulo, L., et al., [83] | Binds to allosteric sites, altering enzyme function without competing with urea | *In vitro* |
|  | Synthetic Allosteric Modulators: Benzimidazole-based inhibitors | Brishty, S.R., et al., [84] | Binds to sites other than the active site, altering structure/function | *In vitro* |
| **Class 8:** Emerging strategies. | Nanomedicine: Silver nanoparticles (AgNPs). | Pop, R., et al., [86] | conjugating to bacterial cell membranes, inhibiting cellular respiration, degrading key respiratory enzymes, inducing cell wall rupture, and increasing membrane permeability via pore formation, leading to cell death. | *In vitro* |
|  | Nanomedicine: Zinc Oxide Nanoparticles (ZnO-NPs). | Cerri, G., et al. [88] | Zn²⁺ ions compete with nickel at active site; they disrupt membranes | *In vitro* |
|  | Nanomedicine: Gold Nanoparticles (AuNPs). | Asgari, S., et al [89] | Binds to active site, often combined with antibiotics | *In vitro* |
|  | Gene Therapy. | Debowski, A.W., et al., [39] | The gene silencing strategy employs  a genetic system (tetracycline-inducible) that allows for controlled expression of the urease gene in *H. pylori*. Silencing the gene inhibits the development of the urease enzyme, thereby eliminating its activity | *In vitro* |
|  | Gene Therapy: Small-interfering RNAs (siRNAs)-Based Gene Silencing (ureB, cagA) | Motamedi, H., et al., [40] | degrade the mRNA of genes encoding key virulence factors in *H.* *pylori*, such as ureB (a subunit of urease) and cagA (associated with gastric cancer development). | *In vitro* |
| Novel synthetic compound | Disulfides (N-Arylacetamide Disulfides). | Liu, M.L., et al., [90] | Reversible, mixed inhibition; binds without covalent modification | *In vivo* |
|  | Sulfonates &amp; Sulfamates (Imidazo[2,1-b] thiazole | Shahin, A.I., et al., [91] | Competitive inhibitor; forms interactions with active site residues | *In vitro* |
|  | Thiosemicarbazones | Islam, M., et al. [92] | Covalent interactions with cysteine residues (potentially irreversible) | *In vitro* |
|  | Benzimidazoles | Zaman, K., et al. [93] | Interactions with nickel ions or amino acid residues, leading to strong inhibition. | *In vitro* |
|  | Bi-heterocyclic Bi-amides (Thiazole-Oxadiazole Derivatives). | Abbasi, M.A., et al. [94] | Acts as a competitive inhibitor, directly binding to the enzyme's active site. Molecular docking shows interactions with key active site residues. | *In vitro*, Molecular Docking |
|  | UreG inhibitors | Yang, X., et al., [95] | UreG is a helper protein that enables urease to function in bacteria such as *H. pylori*. Its main job is to deliver nickel, a necessary metal, into the urease enzyme to activate it, a process called maturation. The strategy of UreG inhibitors is to stop this helper protein from working. | *In vitro* |
| Others  (mentioned in the therapeutic use section | 2-Mercaptoacetamide (2-MA) | Badal, B.D. and J.S. Bajaj [101] | Mimics urea or forms a tetrahedral intermediate; blocks urease & biofilm | Experimental, In silico |
|  | Dimethyl Sulfoxide (DMSO) | Evstafeva, D. [25] | Noncompetitive inhibition of urease activity in whole cells | *In vitro* |
|  | Probiotic (Lactobacillus) | Szczerbiec, D. [102] | Lactic acid modulates urease activity; it inhibits crystallization | *In vitro* & In silico |
|  | Combination (TA, NaF, AHA) | Haq, M.A. [103] | Synergistic reduction of ureolytic activity, delaying ammonia production | *In vitro* |
| **Abbreviations**: **EDTA**: Ethylenediaminetetraacetic acid. **Pal**: Palmatine. **MOSs**: Metal-organic salts. **AgNPs**: Silver nanoparticles. **ZnO-NPs**: Zinc Oxide Nanoparticles. **siRNAs**: Small-interfering RNAs. **DMSO**: Dimethyl Sulfoxide. **2-MA**: 2-Mercaptoacetamide. This table is generated by authors. | | | | |
