# Supplementary material for: Urease inhibitors: current advances, therapeutic potentials, and clinical challenges
Source: Future Med Chem. 2026 Jun 15;18(14):1907–23. doi: 10.1080/17568919.2026.2684497 (PMC13557569; doi:10.1080/17568919.2026.2684497)
Supplement: Supplementary file 2 [file IFMC_A_2684497_SM8840.docx]

**Representative Structures of Common Urease Inhibitors**

**Metal-based complexes:**

Representative structures of metal-based complexes including Cu, Zn, and Fe coordination geometries.

3.1.2. Schiff base copper metal complexes

3.1.3. Schiff base nickel metal complexes.

3.1.4.Schiff base zinc metal complexes

3.1.5. Schiff base cobalt metal complexes

3.1.7. Acetohydroxamic acid (AHA)

3.1.8.	Phosphorodiamidates (PPD)

3.1.9.	Ethylenediaminetetraacetic acid (EDTA)

3.2.1.	Thioureas:

3.2.2.	Benzimidazole derivatives

3.2.3.	Fluoroquinolone derivatives:

Ciprofloxacin

3.2.4.	Sulfonamides

The sulfonamide group (-SO₂NH₂)

3.3.1.	Flavonoids

Eriodictyol

3.3.2.	Berberine

3.3.3.	Ursolic acid

3.3.4.	Sulforaphane (from cruciferous vegetables)

3.3.5.	Palmatine (Pal).

3.4.	Class 4: Peptide-based inhibitors

3.5.1.	Metal-organic salts

3.5.2.	Polymer-Based and Dual-Targeting Hybrids Molecules:

This is an example of (Ciprofloxacin-Thiourea)

3.6.1.	Boronic Acids:

Phenylboronic Acid

3.6.2.	Phosphoramide Derivatives:

3.6.3.	Bis (N-methylaminomethyl)phosphinic acid

3.6.4.	Phenylphosphorodiamidate (PPD), N-n-butylthiophosphorictriamide (NBPT) and N-n-butylphosphorictriamide (NBPTO)

3.6.5.	Uroofamide (N-(diaminophosphinyl)-4-uorobenzenamide).

Fluorofamide

3.9.1.	Disulfides (N-Arylacetamide Disulfides).

3.9.2.	Imidazo[2,1-b] thiazole Scaffold

3.9.3.	Thiosemicarbazones: N4-substituted Thiosemicarbazone

3.9.4.	Benzimidazoles: This molecule is 2-Amino Benzimidazole

3.9.5.	Bi-heterocyclic Bi-amides (Thiazole-Oxadiazole Derivatives):
